# Numerical simulations of multicomponent ecological models with adaptive methods

**DOI:** 10.1186/s12976-016-0027-4

**Published:** 2016-01-08

**Authors:** Kolade M. Owolabi, Kailash C. Patidar

**Affiliations:** Department of Mathematics and Applied Mathematics, University of the Western Cape, Bellville, 7535 Cape Town South Africa

**Keywords:** Coexistence, Competitive, Exponential time differencing, Predator-prey, Mutualism, Multistep methods, Reaction-diffusion systems, Stability analysis, Primary 70K05, 65M20, Secondary 70K20, 74H65

## Abstract

**Background:**

The study of dynamic relationship between a multi-species models has gained a huge amount of scientific interest over the years and will continue to maintain its dominance in both ecology and mathematical ecology in the years to come due to its practical relevance and universal existence. Some of its emergence phenomena include spatiotemporal patterns, oscillating solutions, multiple steady states and spatial pattern formation.

**Methods:**

Many time-dependent partial differential equations are found combining low-order nonlinear with higher-order linear terms. In attempt to obtain a reliable results of such problems, it is desirable to use higher-order methods in both space and time. Most computations heretofore are restricted to second order in time due to some difficulties introduced by the combination of stiffness and nonlinearity. Hence, the dynamics of a reaction-diffusion models considered in this paper permit the use of two classic mathematical ideas. As a result, we introduce higher order finite difference approximation for the spatial discretization, and advance the resulting system of ODE with a family of exponential time differencing schemes. We present the stability properties of these methods along with the extensive numerical simulations for a number of multi-species models.

**Results:**

When the diffusivity is small many of the models considered in this paper are found to exhibit a form of localized spatiotemporal patterns. Such patterns are correctly captured in the local analysis of the model equations. An extended 2D results that are in agreement with Turing typical patterns such as stripes and spots, as well as irregular snakelike structures are presented. We finally show that the designed schemes are dynamically consistent.

**Conclusion:**

The dynamic complexities of some ecological models are studied by considering their linear stability analysis. Based on the choices of parameters in transforming the system into a dimensionless form, we were able to obtain a well-balanced system that is biologically meaningful. The accuracy and reliability of the schemes are justified via the computational results presented for each of the diffusive multi-species models.

## Background

The study of reaction-diffusion problems in ecological context have gained a huge amount of scientific interest, due to their practical relevance and emergence of some interesting phenomena such as spatial patterns, oscillating solutions, phase planes, chaotic behaviours and multiple steady states to mention a few. The most popular and well-known predator-prey model is named after the two scientists, Alfred Lotka (1880–1949) and Vito Volterra (1860–1940). Lotka and Volterra in their earlier research work apply the model to address the interacting population systems called predator–prey. Our numerical study in this paper is aimed at reflecting the types of interactions which we describe as predation (a process where one species of organisms called predator depends solely on the other known as the prey, for survival), competition (a situation whereby two or more different species of organisms struggle for the available resources; definitely, we expect the growth rate of each population to decrease) and lastly, the mutualism or symbiosis (organisms coexist without negatively affecting each other and hence, species growth rate is increased) [2, 11, 12, 23, 32, 33, 37, 39, 43, 48, 49].

A lot of research attention has been devoted to the study of population dynamics with regards to ecological interactions over the past few decades. Such studies include the predator-prey system that describes the situation in which the existence of the species called the predator depends solely on the other species called the prey. The predator-prey system has received tremendous attraction over the years but has been represented mainly in terms of ordinary differential equations, which modelled the species distribution. The Dynamics of the Lotka-Volterra predator-prey model are quite interesting. However, this model is structurally unstable since a small perturbation of the equations often results to a drastic change in the dynamical system. For this reason, the presence of diffusion mechanism takes place though it changes the behavior of the whole model to coupled partial differential equations, called reaction-diffusion system. With the introduction of diffusion, the analysis of the whole system remain tactical in the literature [46, 47], and therefore, numerical approximations are quite often used to simulate these types of models.

Predator-prey systems have been studied by many researchers in various forms. For instance, in bacteria ecology, computer simulations of complex spatiotemporal patterns [4, 11] of *Bacillus subtilis* based on stochastic models [22] and deterministic models [29], Allee effect of patchy invasion on predator-prey dynamics [1, 3, 5, 7, 13, 39]. The diffusive predator-prey systems have also been studied extensively, see, [11, 17, 26, 27, 31, 38, 43]. Moreover, Wang et al. [46] investigated the spatial pattern formation of a predator-prey system with prey-dependent functional response of Ivlev type and reaction-diffusion whereas the analysis of predator-prey systems showing the Holling type II functional response is examined in Garvie and Trenchea [12].

Another type of inter-species interaction is given by the competition. Competitive species models or community models further describe a situation where consumers share some resources that can affect their rate of production. Many ecologist, however, put greater weight on competition which was thought to play a predominant role over the years in structuring ecological communities. Notwithstanding, there is a classical model of competition due to Lotka [24, 25] and Volterra [44, 45]. The Lotka-Volterra competition model is an interference model where two species are assumed to diminish each other's per capita growth rate by direct interference. It is usually assumed in this model that each species has a different population of different sizes that grow logistically in absence of each other and that each has a per capita growth rate that decreased linearly with the population size with their own intrinsic growth rate and carrying capacity. Mathematically, the simplest and instructive case is described by a system of two coupled-reaction diffusion equations. The system of two competing species in just one-dimensional space has attracted a lot of attentions, see [10, 14, 48] for details. Some of the evolution processes here are characterized owing to the fact that certain moments of time they experience a sudden change of state. To this end, we additionally consider a general case of $n$ competing species that is less investigated and still poorly understood for case *n* ≥ 2. Among the few works done when *n* > 2 include [37, 40, 43].

In mutualistic systems, organisms are found to evolve together. The existence of one has no negative effect on the other, each is part of the other's environment and co-exist, and they make use of each other in such a way that both organisms are benefited. Mutualism has not been given as much attention as predation and competition. Readers are referred to [20, 23] for a thorough review of the natural history of mutualism. Community invasion models have an issue of significant importance in the contemporary study of biological and ecological systems which have drawn the attention of both theorists and ecologists since the foundation work of Holt [18]. Despite a considerable achievements recorded in the field of population dynamics modeling the interaction of a multi-species community, so many challenging and open problems that are of great ecological importance are yet to be addressed.

### Mathematical analysis of the main equations

In this work, our major attention is on the two-variable reaction-diffusion systems. We shall adapt linear stability analysis method to discuss the general two species dynamics. Let *u* and *v* be the variables representing the two species of the Lotka-Volterra predator-prey type. In the convention here, *v* is the predator, while *u* represents the prey.

The most relevant and general two-species reaction-diffusion system is formulated as

1 $$ \left.\begin{array}{l}\frac{\partial u}{\partial t}=Du\frac{\partial^2u}{\partial {x}^2}+f\left(u,v\right),\\ {}\frac{\partial v}{\partial t}=Dv\frac{\partial^2v}{\partial {x}^2}+g\left(u,v\right),\end{array}\right\} $$

subject to zero-flux boundary conditions on a closed interval, say [0, *L*]

2 $$ \left.\begin{array}{l}\frac{\partial u}{\partial x}\left(0,t\right)=\frac{\partial u}{\partial x}\left(L,t\right)=0,\\ {}\frac{\partial v}{\partial x}\left(0,t\right)=\frac{\partial v}{\partial x}\left(L,t\right)=0.\end{array}\right\} $$

We assume that the point $\left(\widehat{u},\widehat{v}\right)$ is stable equilibrium state of the homogeneous system

3 $$ \frac{du}{dt}=f\left(u,v\right),\kern0.48em \frac{du}{dt}=g\left(u,v\right), $$

that is $f\left(\widehat{u},\widehat{v}\right)=0,\kern0.48em g\left(\widehat{u},\widehat{v}\right)=0$. Stability of the steady states for general two-variable system can be represented by the Jacobian

4 $$ J=\left(\begin{array}{l}{J}_{11}\kern0.36em {J}_{12}\\ {}{J}_{21}\kern0.36em {J}_{22}\end{array}\right)=\left(\begin{array}{l}\frac{\partial F}{\partial u}\left|{}_{\left(\widehat{u},\widehat{v}\right)}\right.\kern0.6em \frac{\partial F}{\partial v}\left|{}_{\left(\widehat{u},\widehat{v}\right)}\right.\\ {}\frac{\partial G}{\partial u}\left|{}_{\left(\widehat{u},\widehat{v}\right)}\right.\kern0.6em \frac{\partial G}{\partial v}\left|{}_{\left(\widehat{u},\widehat{v}\right)}\right.\end{array}\right) $$

Which leads to characteristic equation of the form *λ*^2^ − *trJλ* + det *J* = 0, where

5 $$ trJ={J}_{11}+{J}_{22}<0,\kern0.6em  \det J={J}_{11}{J}_{22}-{J}_{12}{J}_{21}>0. $$

To examine the stability of the uniform steady state $\left(\widehat{u}(x),\widehat{v}(x)\right)=\left(\widehat{u},\widehat{v}\right)$, we carry out the linear stability analysis in the spirit of Allen [2], Mendez et al. [28] and Murray [32, 33], we obtain

6 $$ \left.\begin{array}{l}u\left(x,t\right)\widehat{u}+{u}_0 \cos (kx){e}^{\lambda_kt},\\ {}v\left(x,t\right)\widehat{v}+{v}_0 \cos (kx){e}^{\lambda_kt},\end{array}\right\} $$

where *λ*_*k*_, the growth rates and the modes cos(*kx*) are the roots of polynomial

7 $$ \det \left(J-D-{I}_n{\lambda}_k\right)=\left(\begin{array}{l}{J}_{11}-{D}_u{k}^2-{\lambda}_k\kern1.8em {J}_{12}\\ {}\kern1.68em {J}_{21}\kern1.68em {J}_{22}-{D}_v{k}^2-{\lambda}_k\end{array}\right)=0, $$

which corresponds to a polynomial

8 $$ {\lambda}_k^2+{\varPhi}_1{\lambda}_k+{\varPhi}_2 $$

representing the dispersion relation, with

$$ \begin{array}{l}{\varPhi}_1=\left({D}_u+{D}_v\right){k}^2-trJ,\\ {}{\varPhi}_2=\left({J}_{11}-{D}_u{k}^2\right)\left({J}_{22}-{D}_v{k}^2\right)-{J}_{12}{J}_{21}={D}_u{D}_v{k}^4-\left({D}_v{J}_{11}+{D}_u{J}_{22}\right){k}^2+ \det J.\end{array} $$

Known from the stability conditions in (5) that *trJ* < 0, thus

$$ {\varPhi}_1=\left({D}_u+{D}_v\right){k}^2-trJ<0,\forall k. $$

Which shows that the uniform steady state of (1) cannot undergo an oscillatory instability (or wave bifurcation) to a standing wave pattern.

A Turing instability corresponds to ${\lambda}_{k_{trJ}}=0$ for *k*_*trJ*_ ≠ 0. That is, with *Φ*_2_ = 0, results to (*J*_11_ − *D*_*u*_*k*^2^)(*J*_22_ − *D*_*v*_*k*^2^) − *J*_12_*J*_21_ = 0 or

9 $$ {k}^4-\left(\frac{J_{11}}{D_u}+\frac{J_{22}}{D_v}\right){k}^2+\frac{ \det J}{D_u{D}_v}=0 $$

For the roots of (9) to be positive,

10 $$ {D}_v{J}_{11}+{D}_u{J}_{22}>0 $$

is a necessary but not sufficient condition for the Turing instability to occur. With reference to conditions in (5), Turing instability can occur if the diffusion coefficient *D*_*u*_ ≠ *D*_*v*_ and if the matrix elements *J*_11_ and *J*_22_ have opposite sign. So, Turing instabilities occur only in either pure or cross activator-inhibitor dynamical system.

The system (3) is of the *pure* Lotka-Volterra type if the Jacobian agrees with the structure of the form

11 $$ J=\left(\begin{array}{l}+\kern0.6em -\\ {}+\kern0.6em -\end{array}\right),\kern1em \mathrm{f}\mathrm{o}\mathrm{r}\kern0.5em {J}_{11}>0,\;{J}_{22}<0,\;{J}_{12}<0,\;{J}_{21}>0 $$

Again, it is of the *cross* Lotka-Volterra type if the Jacobian has the structure

12 $$ J=\left(\begin{array}{l}+\kern0.6em +\\ {}-\kern0.6em -\end{array}\right),\kern1em \mathrm{f}\mathrm{o}\mathrm{r}\kern0.5em {J}_{11}>0,\;{J}_{22}<0,\;{J}_{12}>0,\;{J}_{21}<0 $$

Clearly from systems (11) or (12), we have *J*_11_ > 0, *J*_22_ < 0 which together with *D*_*v*_*J*_11_ + *D*_*u*_*J*_22_ > 0 indicates that Turing instability can occur only if |*J*_22_| > *J*_11_ since *trJ* < 0 and *J*_12_*J*_21_ < 0 for det *J* > 0. If we let *Θ*_*D*_ = *D*_*v*_/*D*_*u*_ be the diffusion coefficients ratio, we can easily obtain from (10) that *Θ*_*D*_ > *J*_22_/*J*_11_ > 1. The indication here is that, for Turing instability to take place, the inhibitor must diffuse faster than the activator.

By rewriting (9) in the form

$$ {k}^4+{\varPsi}_1{k}^2+{\varPsi}_2=0, $$

where

$$ {\varPsi}_1=-\left(\frac{J_{11}}{D_u}+\frac{J_{22}}{D_v}\right),\kern0.84em {\varPsi}_2=\frac{ \det J}{D_u{D}_v}, $$

we have the roots of equation (9) given by

$$ {k}_{1,2}^2=-\frac{\varPsi_1}{2}\pm \frac{\sqrt{\varPsi_1^2-4{\varPsi}_2}}{2}, $$

provided *Ψ*_1_ < 0 and condition (10) is satisfied. So, Turing instability occurs for (10) to have a double root, that is, if *Ψ*_1_^2^ − 4*Ψ*_2_ = 0.

In conclusion, the uniform steady state of the reaction-diffusion system (1), $\left(\widehat{u}(x),\widehat{v}(x)\right)=\left(\widehat{u},\widehat{v}\right)$, that satisfy the stability conditions in (5) will be unstable in the presence of diffusion (called diffusion driven-instability) if *Ψ*_1_ < 0, that is, *D*_*v*_*J*_11_ + *D*_*u*_*J*_22_ > 0, and *Ψ*_1_^2^ − 4*Ψ*_2_ > 0, that is, (*D*_*v*_*J*_11_ + *D*_*u*_*J*_22_)^2^ > 4*D*_*u*_*D*_*v*_ det *J*, with the band of unstable modes

$$ \left(\frac{-{\varPsi}_1-\sqrt{\varPsi_1^2-4{\varPsi}_2}}{2}\right)<{k}^2<\left(\frac{-{\varPsi}_1+\sqrt{\varPsi_1^2-4{\varPsi}_2}}{2}\right). $$

A good research focus has to be given to the numerical simulation of multi-species dynamics in more than one dimensional space which has received little attention in the literature. We simulated a class of biological systems that lead to the evolution of traveling waves and formation of chaotic and spatiotemporal patterns arising in the context of mathematical ecology. Though, simulations that are based on the use of conventional methods in two-dimensions are found to be time consuming. As a result, consideration is given to the design and method of implementing a viable numerical scheme that can handle a class of multi-component reaction-diffusion problems efficiently.

### Numerical methods

Many systems of nonlinear time dependent reaction-diffusion problems of partial differential equations that are of physical interest are written in the compact form

13 $$ \frac{\partial w}{\partial t}=D{\nabla}^2w+N\left(w,t\right) $$

where *D* > 0 is the diffusion coefficient, ∇^2^*w* = (∂^2^*w*/∂*x*^2^ + ∂^2^*w*/∂*y*^2^) is the two-dimensional Laplacian operator that represents the linear term, and *N* is nonlinear function of *w* and *t*. By following [34–36], we discretize the spatial domain by mesh (*x*_*i*_, *y*_*j*_) = (*T*_1_ + *i* × *h*_*x*_, *T*_1_ + *j* × *h*_*y*_) where *h*_*x*_ = (*T*_2_ − *T*_1_)/(*N*_*x*_ + 1), *h*_*y*_ = (*T*_2_ − *T*_1_)/(*N*_*y*_ + 1), for 0 ≤ *i* ≤ *N*_*x*_ + 1, 0 ≤ *j* ≤ *N*_*y*_ + 1. We approximate the second-order derivatives by the fourth-order central difference operators

$$ \frac{\partial^2w}{\partial {x}^2}=\frac{-{w}_{i-2,j}+16{w}_{i-1,j}-30{w}_{i,j}+16{w}_{i+1,j}-{w}_{i+2,j}}{12{h}_x^2}={L}_1, $$

$$ \frac{\partial^2w}{\partial {y}^2}=\frac{-{w}_{i,j-2}+16{w}_{i,j-1}-30{w}_{i,j}+16{w}_{i,j+1}-{w}_{i,j+2}}{12{h}_y^2}={L}_2, $$

and

$$ w={\left(\begin{array}{cccc}\hfill {w}_{i,j}\hfill & \hfill {w}_{i,j+1}\hfill & \hfill \cdots \hfill & \hfill {w}_{i,{N}_y+1}\hfill \\ {}\hfill {w}_{i+1,j}\hfill & \hfill {w}_{i+1,j+1}\hfill & \hfill \cdots \hfill & \hfill {w}_{i+1,{N}_y+1}\hfill \\ {}\hfill \vdots \hfill & \hfill \vdots \hfill & \hfill \vdots \hfill & \hfill \vdots \hfill \\ {}\hfill {w}_{N_x,j}\hfill & \hfill {w}_{N_x,j+1}\hfill & \hfill \cdots \hfill & \hfill {w}_{N_x,{N}_y+1}\hfill \end{array}\right)}_{N_n\times {N}_y+1} $$

for *i* = 1, 2, …, *N*_*x*_ and *j* = 1, 2, …, *N*_*y*_ + 1. The discretized form of (13) lead to a coupled system ordinary differential equations (ODEs)

14 $$ \frac{dw}{dt}=D\left({L}_1+{L}_2\right)w+N\left(w,t\right) $$

where *L*_1_, *L*_2_ ∈ *L* and *w* = *w*(*u*, *v*).

Exponential integrators separate the linear term involving *L*, which is solved exactly by a matrix exponential, from the nonlinear term. The theory of numerical methods for the time integration of semi-linear problems has been proposed by the application of the exponential methods. Cox and Matthews [6] presented derivation of exponential time differencing (ETD) methods. Few years later, a modification of the ETD Runge-Kutta methods of Cox and Matthews was made by Kassam and Trefethen [21], and it is from their paper that we present some details of the scheme. A new algorithm for the implementation of the exponential methods has been discussed in [9], that the algorithm evaluates the operator by the exponential methods with a quadrature formula that converges. Hochbruck and Ostermann [15] discussed further on the class of explicit multistep exponential and explicit exponential Runge-Kutta methods. In this paper, we use both the fourth-order exponential time differencing Runge-Kutta method [6, 21] and the fourth-order exponential multistep method of Adams-type [6, 16].

### Exponential time differencing method

We present a brief introduction to the derivation of exponential time differencing Runge-Kutta and multistep methods of Adams-type along with their stability regions. Details of their derivations can be found in [6, 16] and the references therein. The exponential time differencing idea, applied here for the *u* component, involves the use of the integrating factor, *e*^− *L t*^. We multiply equation (14) by this factor and then integrate it over a time-step to obtain

15 $$ w\left({t}_{n+1}\right)=w\left({t}_n\right){e}^{L\varDelta t}+{e}^{L\varDelta t}{\displaystyle \underset{0}{\overset{\varDelta t}{\int }}{e}^{-L\tau }N\left(w\left({t}_n+\tau \right),{t}_n+\tau \right)d\tau } $$

This equation is known to be exact [6, 21]. Various ETD schemes come from how one approximates the integral on the right hand side in the above equation. The article by Cox and Matthews [6] contains various approximations to the integral. They first presented the sequence of recurrence formulae that give higher-order approximations of a multistep method of Adams-type. In their work, a general ETD scheme of order-*s* was proposed as

16 $$ {w}_{n+1}={w}_n{e}^{L\varDelta t}+\varDelta t{\displaystyle \sum_{j=0}^{s-1}{g}_j}{\displaystyle \sum_{k=0}^j{\left(-1\right)}^k}\left(\begin{array}{c}\hfill j\hfill \\ {}\hfill k\hfill \end{array}\right){N}_{n-k}. $$

The coefficients g_*j*_ are obtained by the recurrence relation

17 $$ \begin{array}{l}L\varDelta t{g}_0={e}^{L\varDelta t}-1,\\ {}L\varDelta t{g}_{j+1}+1={g}_j+\frac{1}{2}{g}_{j-1}+\frac{1}{3}{g}_{j-2}+\cdots +\frac{1}{j+1}{g}_0={\displaystyle \sum_{k=0}^j\frac{1}{j+1-k}{g}_k}.\end{array} $$

By setting *s* = 4 in the explicit integrating formula (16), we obtain the fourth-order ETD scheme of Adams-type

18 $$ {w}_{n+1}={w}_n{e}^{L\varDelta t}+\left({\varTheta}_1{N}_n-{\varTheta}_2{N}_{n-1}+{\varTheta}_3{N}_{n-2}-{\varTheta}_4{N}_{n-3}\right)/\left(6{L}^2\varDelta {t}^3\right), $$

where

$$ \begin{array}{l}{\varTheta}_1=\left(6{L}^3\varDelta {t}^3+11{L}^2\varDelta {t}^2+12L\varDelta t+6\right){e}^{L\varDelta t}-24{L}^3\varDelta {t}^3-26{L}^2\varDelta {t}^2-18L\varDelta t-6,\\ {}{\varTheta}_2=\left(18{L}^2\varDelta {t}^2+30L\varDelta t+18\right){e}^{L\varDelta t}-36{L}^3\varDelta {t}^3-57{L}^2\varDelta {t}^2-48L\varDelta t-18,\\ {}{\varTheta}_3=\left(6{L}^2\varDelta {t}^2+24L\varDelta t+18\right){e}^{L\varDelta t}-24{L}^3\varDelta {t}^3-42{L}^2\varDelta {t}^2-42L\varDelta t-18,\\ {}{\varTheta}_4=\left(2{L}^2\varDelta {t}^2+6L\varDelta t+6\right){e}^{L\varDelta t}-6{L}^3\varDelta {t}^3-11{L}^2\varDelta {t}^2-12L\varDelta t-6,\end{array} $$

denoted in this paper as ETDADAMS4.

Similarly, Cox and Matthews derived a set of ETD schemes that are based on Runge-Kutta time-stepping, which they call ETDRK schemes. We only use the fourth-order scheme which we denoted as ETDRK4 in this paper. On setting *s* = 4 again in (16), we have the ETDRK4 formula

19 $$ \begin{array}{l}{w}_{n+1}={w}_n{e}^{L\varDelta t}+{N}_n\left[-4-L\varDelta t+{e}^{L\varDelta t}\left(4-3L\varDelta t+{L}^2\varDelta {t}^2\right)\right]\\ {}\kern1.2em +2\left(N\left({a}_n,{t}_n+\varDelta t/2\right)+N\left({b}_n,{t}_n+\varDelta t/2\right)\right)\left[2+L\varDelta t+{e}^{L\varDelta t}\left(-2+L\varDelta t\right)\right]\\ {}\kern1.2em +N\left({c}_n,{t}_n+\varDelta t\right)\left[-4-3L\varDelta t-{L}^2\varDelta {t}^2+{e}^{L\varDelta t}\left(4-L\varDelta t\right)\right]/{L}^3\varDelta {t}^2,\end{array} $$

where

$$ \begin{array}{l}{a}_n={w}_n{e}^{L\varDelta t/2}+\left({e}^{L\varDelta t/2}-I\right){N}_n/L,\\ {}{b}_n={w}_n{e}^{L\varDelta t/2}+\left({e}^{L\varDelta t/2}-I\right)N\left({a}_n,{t}_n+\varDelta t/2\right)/L,\\ {}{c}_n={w}_n{e}^{L\varDelta t/2}+\left({e}^{L\varDelta t/2}-I\right)\left(2N\left({b}_n,{t}_n+\varDelta t/2\right)-{N}_n\right)/L.\end{array} $$

To circumvent the pronounced vulnerability of error cancelations in the higher-order ETDADAMS4 and ETDRK4 schemes [21], and to generalize the ETD schemes to non-diagonal problems, modified schemes are proposed with the aid of complex contour integral

20 $$ \varphi (L)=\frac{1}{2\pi i}{\displaystyle {\int}_{\varGamma}\varphi (t){\left(tI-L\right)}^{-1}dt}, $$

to evaluate the coefficients of these schemes. Further details on derivations and applications of ETD Adams-type and ETD Runge-Kutta methods can be found in [6, 21, 41].

### Stability analysis

We investigate the stability of the fourth-order ETDADAMS4 (18) and ETDRK4 (19) schemes by linearizing the nonlinear autonomous system [8, 19]

21 $$ \frac{dw(t)}{dt}=Lw(t)+N\left(w(t)\right), $$

with *N(w(t))* the nonlinear part. We suppose that there exists a fixed point *w*_*0*_ such that *Lw*_*0*_ *+ N(w*_*0*_*)* = 0. Linearizing about this fixed point, we obtain

22 $$ \frac{dw(t)}{dt}=Lw(t)+\lambda w(t), $$

where *w(t)* is now the perturbation of *u*_*0*_ and *λ* = *N*′(*w*_0_) is a diagonal or a block diagonal matrix containing the eigenvalue of *N*. In an attempt to keep the fixed point u0 stable, we require that Re(*L* + *λ*) < 0, for all *λ*. It is naturally important for a numerical method to satisfy this property so as to cover as much dynamics as possible.

When applying ETDADAMS4 (18) to the linearized problem (22), a polynomial equation of the order-four in *r* is obtained in the form

23 $$ {w}_4{r}^4+{w}_3{r}^3+{w}_2{r}^2+{w}_1r+{w}_0=0, $$

where

$$ \begin{array}{l}{w}_0=\left[\left(2{y}^2+6y+6\right){e}^y-6{y}^3-11{y}^2-12y-6\right]x,\\ {}{w}_1=\left[\left(-9{y}^2-24y-18\right){e}^y+24{y}^3+42{y}^2+42y+18\right]x,\\ {}{w}_2=\left[\left(18{y}^2+30y+12\right){e}^y-36{y}^3-57{y}^2-48y-18\right]x,\\ {}{w}_3=-6{y}^6{e}^y+\left[\left(-6{y}^3-11{y}^2-12y-6\right){e}^y+24{y}^3+26{y}^2-18y+6\right]x,\\ {}{w}_4=6{y}^4.\end{array} $$

**Fig. 1:**
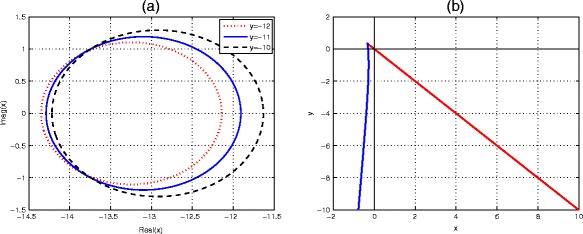
Stability regions of the ETDADAMS4 scheme (18). We plot the region stability of the ETDADAMS4 scheme both in (**a**) the complex plane *x* and (**b**) the real *(x, y)* plane

In the real *(x, y)* plane, the right-hand boundary for ETDADAMS4 scheme corresponds to substituting *r* = 1 in equations (23) is the line *x + y* = 0. The corresponding left-hand boundary for substituting *r* = −1, also in (23), is given by the curve

24 $$ x=\frac{3{y}^4\left({e}^y+1\right)}{\left(3{y}^3+20{y}^2+36y+24\right){e}^y-45{y}^3-68{y}^2-60y-24} $$

as displayed in Fig. 1.

**Fig. 2:**
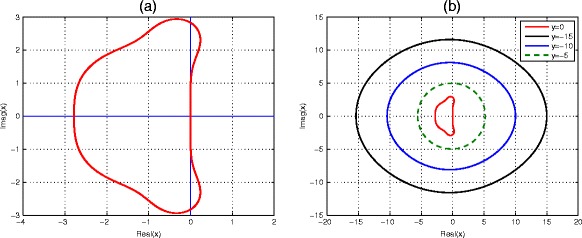
Stability region of the ETDRK4 scheme (19). Boundary of stability regions in the complex *x* plane for the ETDRK4 scheme at (**a**) when *y* = 0, which correspond to the stability regions of the classical fourth-order Runge-Kutta method, and (**b**) shows the curve of ETDRK4 at some negative values of *y* = −15, −10, −5, from outer to the inner curves. The innermost curve corresponds to the stability region of (**a**) at *y* = 0

In a similar fashion, the application of ETDRK4 method (19) to the linearized problem (22) leads to a recurrence relation

25 $$ r=\frac{w_{n+1}}{w_n}={L}_0+{L}_1x+{L}_2{x}^2+{L}_3{x}^3+{L}_4{x}^4, $$

where

$$ \begin{array}{l}{L}_0={e}^y\\ {}{L}_1=-\frac{4}{y^3}+\frac{8{e}^{y/2}}{y^3}-\frac{8{e}^{3y/2}}{y^3}+\frac{4{e}^{2y}}{y^3}-\frac{1}{y^2}+\frac{4{e}^{y/2}}{y^2}-\frac{6{e}^y}{y^2}+\frac{4{e}^{3y/2}}{y^2}-\frac{e^{2y}}{y^2}\\ {}{L}_2=-\frac{8}{y^4}+\frac{16{e}^{y/2}}{y^4}-\frac{16{e}^{3y/2}}{y^4}+\frac{8{e}^{2y}}{y^4}-\frac{5}{y^3}+\frac{12{e}^{y/2}}{y^3}-\frac{10{e}^y}{y^3}+\frac{4{e}^{3y/2}}{y^3}\\ {}\kern1.2em -\frac{e^{2y}}{y^3}-\frac{1}{y^2}+\frac{4{e}^{y/2}}{y^2}-\frac{e^{y/2}}{y^2}\\ {}{L}_3=\frac{4}{y^5}-\frac{16{e}^{y/2}}{y^5}+\frac{16{e}^y}{y^5}+\frac{8{e}^{3y/2}}{y^5}-\frac{20{e}^{2y}}{y^5}+\frac{8{e}^{5y/2}}{y^5}+\frac{2}{y^4}-\frac{10{e}^{y/2}}{}\\ {}\kern1.32em +\frac{16{e}^y}{y^4}-\frac{12{e}^{3y/2}}{y^4}+\frac{6{e}^{2y}}{y^4}-\frac{2{e}^{5y/2}}{y^4}-\frac{2{e}^{y/2}}{y^3}+\frac{4{e}^y}{y^3}-\frac{2{e}^{3y/2}}{y^3}\\ {}{L}_4=\frac{8}{y^6}-\frac{24{e}^{y/2}}{y^6}+\frac{16{e}^y}{y^6}+\frac{16{e}^{3y/2}}{y^6}-\frac{24{e}^{2y}}{y^6}+\frac{8{e}^{5y/2}}{y^6}+\frac{6}{y^5}-\frac{18{e}^{y/2}}{y^5}\\ {}\kern1.32em +\frac{20{e}^y}{y^5}-\frac{12{e}^{3y/2}}{y^5}+\frac{6{e}^{2y}}{y^5}-\frac{2{e}^{5y/2}}{y^5}+\frac{4}{y^4}-\frac{6{e}^{y/2}}{y^4}+\frac{6{e}^y}{y^4}-\frac{2{e}^{3y/2}}{y^4},\end{array} $$

where *x* = *λh*, *y* = *Lh*. We can define the amplification factor for ETDRK4, *r(x, y)* for *y >* 0. If y = 0, the amplification factor becomes 1 − *x* + *x*^2^/2 − *x*^3^/6 + *x*^4^/24. Hence, we can see that the stability curve of ETDRK4 at *y* = 0 coincides with that of the classical fourth-order Runge-Kutta method, Fig. 2(a). We also see that lim_*x*,*y* → 0_∂_*x*_*r*(*x*, *y*) = − 1 and lim_*x*,*y* → 0_∂_*y*_*r*(*x*, *y*) = − 1. Hence, the absolute value of the amplification factor is given as |*r*(*x*, *y*)| ≤ 1.

The boundary of the stability region can be determined by setting *r* = *e*^*iθ*^, for *θ* ∈ [0, 2*π*]. We plot the stability region in the complex *x* plane and displayed in Fig. 2, where the horizontal and vertical axes represent the real and imaginary of *x*, respectively.

### Numerical examples and results

In this section, numerical methods we discussed above are now applied to the three major classes of the Lotka-Volterra two-species models. In addition, comparison with other adaptive methods are made to justify the effectiveness and accuracy of the present method. A possible extension to two space dimensions is considered, since it is in higher dimensions that most of the ideas reported are of serious value.

### Predator-prey system

It is clear from our introduction that predator-prey models are similar in description to both parasite and parasitoid models. A typical example of predator-prey model [11, 32] is the reaction-diffusion system

26 $$ \left.\begin{array}{l}\frac{\partial U}{\partial T}={D}_1\frac{\partial^2U}{\partial {X}^2}+U\left[\alpha \left(1-\frac{U}{K}\right)-\frac{\gamma V}{U+\delta}\right],\\ {}\frac{\partial V}{\partial T}={D}_2\frac{\partial^2V}{\partial {X}^2}+V\left[\beta \left(1-\frac{hV}{U}\right)\right],\end{array}\right\} $$

where *U* and *V* are the densities of the prey and predator respectively, *D*_1_ > 0 and *D*_2_ > 0 are diffusion coefficients for the prey and predator. *α*, *β*, *γ*, *δ*, *h* and *K* are positive parameters. The term *αU*(1 − *U*/*K*) represents the logistic growth, *α* is the intrinsic growth rate, and *K* the carrying capacity. The term *γV* is the per-capita prey reduction due to consumption by the predator, and *β* describes the intensity of predation.

To reduce the number of parameters in (26), we nondimensionalize the model by re-scaling the variables as

27 $$ u(t)=\frac{U(T)}{K},\kern0.48em v(t)=\frac{hV(T)}{K},\kern0.6em t=\alpha T,\kern0.6em \mu =\frac{\gamma }{h\alpha },\kern0.6em \psi =\frac{\beta }{\alpha },\kern0.6em \varphi =\frac{\delta }{K},\kern0.6em D=\frac{D_2}{D_1} $$

to yield

28 $$ \left.\begin{array}{l}\frac{\partial u}{\partial t}=\frac{\partial^2u}{\partial {x}^2}+u\left(1-u\right)-\frac{\mu u}{u+\varphi }v=f\left(u,v\right),\\ {}\frac{\partial v}{\partial t}=D\frac{\partial^2v}{\partial {x}^2}+\psi v-\frac{\psi {v}^2}{u}=g\left(u,v\right).\end{array}\right\} $$

For the linear stability, we have to analyze the stability criteria of the non-diffusive system [17, 31, 42]. The spatial model (28) has the corresponding non-diffusive systems

29 $$ \left.\begin{array}{l}\frac{du}{\partial t}=u\left(1-u\right)-\frac{\mu u}{u+\varphi }v=f\left(u,v\right),\\ {}\frac{dv}{\partial t}=\psi v-\frac{\psi {v}^2}{u}=g\left(u,v\right),\end{array}\right\} $$

with just three parameters *μ* > 0, *ψ* > 0 and *φ* > 0. There are other choices for the change of variables to put the system in dimensionless form, but we opt for the choice that suits our purpose since the dimensionless groupings used here give relative measures of the effect of dimensional parameters. For instance, ' now becomes the ratio of the linear growth rate of the predator to that of the prey, for *ψ* < 1. We expect the prey to reproduce faster than the predator otherwise the system will go into extinction.

At equilibrium, $f\left(\widehat{u},\widehat{v}\right)=g\left(\widehat{u},\widehat{v}\right)=0$, since the steady state populations *û* and $\widehat{v}$ are solutions of *du/dt = dv/dt* = 0. Hence,

30 $$ \left.\begin{array}{l}\widehat{u}\left(1-\widehat{u}\right)-\frac{\mu \widehat{u}}{u+\varphi}\widehat{v}=0,\\ {}\psi \widehat{v}-\frac{\psi {\widehat{v}}^2}{\widehat{u}}=0.\end{array}\right\} $$

Naturally, for the dynamical system under consideration to be biologically meaningful, we should have both *u* ≥ 0, *v* ≥ 0 at all times. We observe from (30) that the system (28) has three positive steady states $\left(\widehat{u},\widehat{v}\right)$, the two trivial states or saddle points are at point (0, 0) which describes complete extinction of both prey and predator and point (1, 0), which shows that the predator is absent leading to unbounded logistic growth of the prey species. The stationary point $\left(\widehat{u},\widehat{v}\right)$ corresponding to the existence of predator and prey, bearing in mind that for the system under consideration to be biologically meaningful, the parameters must be strictly restricted to the positive quadrants, gives

31 $$ \widehat{u}=\widehat{v}=\frac{\left(1-\mu -\varphi \right)+{\left[{\left(1-\mu -\varphi \right)}^2+4\varphi \right]}^{1/2}}{2}. $$

The stability of the steady or equilibrium states are the singular points in the phase plane of (28). To determine them, we let

32 $$ A=\left(\begin{array}{cc}\hfill \widehat{u}\left[\frac{\mu \widehat{u}}{{\left(\widehat{u}+\varphi \right)}^2}-1\right]\hfill & \hfill \frac{-\mu \widehat{u}}{\widehat{u}+\varphi}\hfill \\ {}\hfill \psi \hfill & \hfill -\psi \hfill \end{array}\right), $$

where *A* is regarded as the community matrix with eigenvalues given by

33 $$ \left|A-\lambda I\right|=0\Rightarrow {\lambda}^2-(trA)\lambda + \det A=0. $$

For stability, we require that Re*λ* < 0. Hence, the necessary and sufficient conditions for linear stability become

34 $$ \left.\begin{array}{l}trA<0\kern0.36em \Rightarrow \kern0.36em \widehat{u}\left[\frac{\mu \widehat{u}-{\left(\widehat{u}+\varphi \right)}^2}{{\left(\widehat{u}+\varphi \right)}^2}\right]<\psi \\ {} \det A>0\kern0.36em \Rightarrow \kern0.36em \frac{{\left(\widehat{u}+\varphi \right)}^2+\mu \left(\widehat{u}+\varphi \right)-\mu \widehat{u}}{{\left(\widehat{u}+\varphi \right)}^2}>0.\end{array}\right\} $$

On substituting *û* in equation (31) provides the stability conditions in terms of the positive parameters *μ*, *ψ* and *φ*.

#### Results in one-dimension for system (26)

Numerical results of the predator-prey system are shown in one-dimension. The initial data and parameter values are given in the figure caption. The initial data are chosen as a result of small perturbations of the steady state solutions *û* and $\widehat{v}$ of the spatially homogeneous system. By varying the choice of parameters lead to different spatial patterns, such as oscillatory smooth, intermittent structure and spatiotemporal patterns. It should be noted that other one-dimensional spatial structures that are not captured here are possible, depending on the choice of the parameter values and initial data.

**Fig. 3:**
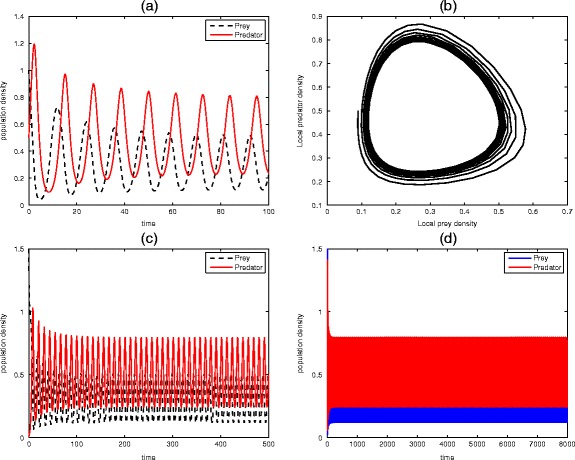
Unrealistic predator-prey system (26). Plots (**a**), (**c**), (**d**) show various periodic solutions of the prey (*u*) and predator (*v*) populations. Parameter values: *μ* = 0.8, *ψ* = 2, *φ* = 0.4, which give a steady state at *û* =1.5, $\widehat{v}$ =0.1 for (**a**) at *t* = 100, (**b**) the local phase plane of the system at *t* = 8000, (**c**) *t* = 500, and (**d**) spatiotemporal oscillations at t = 8000. We expect to see that the prey produces faster than the predator but the case here is otherwise. Take note of the amplitudes

**Fig. 4:**
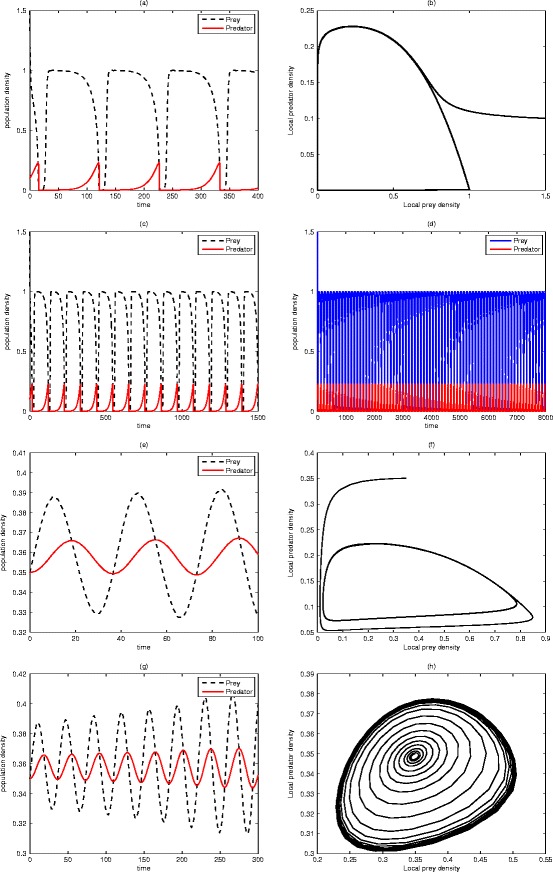
Realistic predator-prey system (26). Typical phase trajectories (**b**), (**f**) and (**h**) of prey-predator system. Time series plots (**a**), (**c**), (**d**), (**e**) and (**g**) show various periodic (oscillatory behaviour) of the prey u and predator v populations. Parameter values: *μ* = 1.5, *ψ* = 0.08, *φ* = 0.01, give a steady state at *û* = 1.5, $\widehat{v}$ = 0.1 for (**a**) at *t* = 400, (**b**) *t* = 7000, (**c**) *t* = 1500, and (**d**) *t* = 8000. By taking *û* = $\widehat{v}$ = 0.35, *μ* = 1, *ψ* = 0.05, *φ* = 0.2, we obtain (**e**) for t = 100, (f) for t = 1000 and (**g**) for t = 300. For (**h**), *μ* = 1.025, t = 8000

Figures 3 and 4 represent the unrealistic and realistic population dynamics of the predator-prey systems. The system with nonlinear part as described in Garvie [11] is quite unrealistic due to the choices of parameters used in transforming the system into a dimensionless form. This shortcoming actually motivates us to choose some appropriate parameters since it is always helpful to write the system in nondimensional form. Nondimensionalisation plays an important role when carefully considered because it reduces the number of parameters by grouping them in a more meaningful manner. So, the system described in Fig. 3 is totally unrealistic as it is prone to danger of extinction of the prey species that would in turn results to total breakdown of the ecosystem since all the predators will die out in absence of food. In Fig. 4, spatiotemporal oscillations arise and population oscillations are transient and regular. It should be noted that due to the formation of spatial pattern, the two species can dynamically coexist.

#### Results in two-dimension for system (26)

**Fig. 5:**
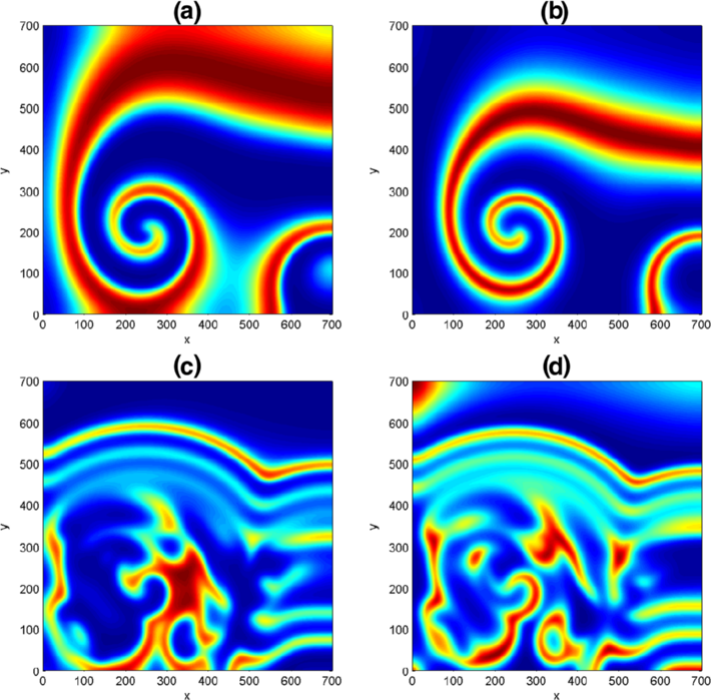
Two-dimensional results for the predator-prey system (26). The first and second columns represent the prey and predator distributions at time *t* = 200 and *t* = 500 for species *u* and *v* respectively

We intend to mimic the two-dimensional results obtained for the predator-prey system in [11, 27], we experiment with the same initial data

35 $$ \left.\begin{array}{l}u\left(x,y,0\right)=\widehat{u}-\left(2\times {10}^{-7}\right)\left(x-0.1y-225\right)\left(x-0.1y-675\right),\\ {}v\left(x,y,0\right)=\widehat{v}-\left(3\times {10}^{-5}\right)\left(x-450\right)-\left(1.2\times {10}^{-4}\right)\left(y-150\right)\end{array}\right\} $$

so as to induce a nontrivial spatiotemporal dynamics of the homogeneous stationary states *û* and $\widehat{v}$. In Fig. 5, numerical simulations was done on a square domain size [0, 700] × [0, 700], with parameter values *D* = 0.1, *μ* = 0.2, *ψ* = 2, *φ* = 0.5 at nontrivial state $\left(\widehat{u},\widehat{v}\right)$ =(6/35, 116/245). As simulation time is increased from *t* = 200 to *t* = 500, the spiral patterns in (a, b) are disjointed and spreads out in the domain to form a stripe-like structures with emergence of some spots underneath. It should be mentioned that if the simulation time is further increased, say to *t* = 1500 and above, there is every tendency of getting a Turing and more complicated spatiotemporal patterns. In addition, we realized that the choice of initial conditions can influence the type of spatiotemporal dynamics of a reaction-diffusion problem in ecosystems.

A close look at the first and second columns in Fig. 5 have revealed that both predator and prey species have a similar distribution. As a result, our pattern formation analysis is henceforth restricted to only one distribution. We also observe in our experiments that increase in domain size actually results to increase in computational time. Henceforth, we choose to simulate with a smaller square domain of size [0, 250] × [0, 250].

### Competitive system

Competition model describes a situation in which two or more species compete for the same (sufficient or insufficient) resources like food, territory or in some way inhibit each other of growth. For simplicity, and by following the approach we used for the predator-prey model, we consider here the two-species Lotka-Volterra competition model

36 $$ \left.\begin{array}{l}\frac{\partial U}{\partial T}={\delta}_1\frac{\partial^2U}{\partial {X}^2}+{\alpha}_1U\left(1-\frac{U}{K_1}-{\beta}_1\frac{V}{K_1}\right),\\ {}\frac{\partial V}{\partial T}={\delta}_2\frac{\partial^2V}{\partial {X}^2}+{\alpha}_2V\left(1-\frac{V}{K_2}-{\beta}_2\frac{U}{K_2}\right),\end{array}\right\} $$

with species *U* and *V* having logistic growth in the absence of the other. The parameters *α*_1_ and *α*_2_ represent their linear birth rates, *β*_1_ and *β*_2_ measure the competitive effect of *V* on *U* and vice versa, *δ*_1_ and *δ*_2_ stand for the diffusion coefficients of species *U* and *V*, and *K*_1_ and *K*_2_ are their respective carrying capacities.

Again, we nondimensionalize (36) by introducing a set of carefully selected dimensionless variables

37 $$ u(t)=\frac{U(T)}{K_1},\kern0.24em v(t)=\frac{V(T)}{K_2},\kern0.24em t={\alpha}_1T,\kern0.24em \mu =\frac{\alpha_2}{\alpha_1},\kern0.24em \varphi ={\beta}_2\frac{K_2}{K_1},\kern0.36em \psi ={\beta}_1\frac{K_1}{K_2},\kern0.36em \delta =\frac{\delta_2}{\delta_1}. $$

As suggested by Medvinsky et al. [27] and Garvie [11], the local stability analysis will always grant a deeper understanding and will provide important information on the choice of parameters for numerical integration. Like the previous case, we continue with the local stability analysis in the absence of diffusion. Using (37) in (36), we obtain

38 $$ \left.\begin{array}{l}\frac{\partial u}{\partial t}=\frac{\partial^2u}{\partial {x}^2}+\left(u-{u}^2-\varphi uv\right)=f\left(u,v\right),\\ {}\frac{\partial v}{\partial t}=\delta \frac{\partial^2v}{\partial {x}^2}+\mu \left(v-{v}^2-\psi uv\right)=g\left(u,v\right).\end{array}\right\} $$

For the linear stability analysis, we consider the case of spatially homogeneous solutions, in which the spatial model (38) is equivalent to the system of ordinary differential equations

39 $$ \left.\begin{array}{l}\frac{du}{\partial t}=\left(u-{u}^2-\varphi uv\right)=f\left(u,v\right),\\ {}\frac{dv}{\partial t}=\mu \left(v-{v}^2-\psi uv\right)=g\left(u,v\right).\end{array}\right\} $$

Here, we regard the steady states and phase plane singularities, *û* and $\widehat{v}$ as the solutions of *f*(*u,v*) *= g*(*u, v*) = 0. This gives four positive equilibrium states,

40 $$ \left(\widehat{u},\widehat{v}\right)=\left(0,0\right),\kern0.24em \left(\widehat{u},\widehat{v}\right)=\left(1,0\right),\kern0.36em \left(\widehat{u},\widehat{v}\right)=\left(0,1\right),\kern0.36em \left(\widehat{u},\widehat{v}\right)=\left(\frac{1-\varphi }{1-\varphi \psi },\frac{1-\psi }{1-\varphi \psi}\right). $$

The good thing is that, all the four steady states exist in the positive quadrant which make the whole process meaningful in the biological and ecological contexts.

The first three stationary states are trivial whereas the last one is non-trivial. The state (0,0) corresponds to total washout state of the two species, the second state (1,0) stands for the existence and extinction of species u and v respectively and the third trivial state (0; 1) indicate that only species v will exist. It is obvious that none of the three trivial states could give a meaningful interpretation about the competition model, therefore, there is the need to explore further the nontrivial equilibrium state $\left(\widehat{u},\widehat{v}\right)$. The points (0,0), (1,0) and (0,1) are all unstable (0,0) is an unstable node, (1,0) and (0,1) are saddle point equilibria. From (39), for *f = g =* 0, we have that (*u* − *u*^2^ − *φuv*) = 0, it follows that either *u =* 0 or 1 − *u* − − *φv* = 0 and also from the second equation, *μ*(*v* − *v*^2^ − *φuv*) = 0 which implies, *μv* = 0 and 1 − *v* − − *φu* = 0.

Now the Jacobian or community matrix for this system evaluated at $\left(\widehat{u},\widehat{v}\right)$ is

41 $$ A={\left(\begin{array}{cc}\hfill 1-2u-\varphi v\hfill & \hfill -\varphi u\hfill \\ {}\hfill -\mu \psi v\hfill & \hfill \mu \left(1-2v-\psi u\right)\hfill \end{array}\right)}_{\left(\widehat{u},\widehat{v}\right)}. $$

The point (0, 0), is unstable since the eigenvalues *λ* obtained from

$$ \left|A-\lambda I\right|=\left|\begin{array}{cc}\hfill 1-\lambda \hfill & \hfill 0\hfill \\ {}\hfill 0\hfill & \hfill \mu -\lambda \hfill \end{array}\right|=0 $$

are *λ*_1,2_ = (1, *μ*). At the point (1, 0), the community matrix *A* gives

$$ \left|A-\lambda I\right|=\left|\begin{array}{cc}\hfill 1-\lambda \hfill & \hfill \varphi \hfill \\ {}\hfill 0\hfill & \hfill \mu \left(1-\psi \right)-\lambda \hfill \end{array}\right|=0. $$

Hence, *λ*_1,2_ = (−1, *μ*(1 − *ψ*)). Therefore, the steady state $\left(\widehat{u},\widehat{v}\right)=\left(1,0\right)$ is stable if *ψ* > 1 and unstable otherwise. In the same manner, we can see that the steady state (0, 1) has the community matrix *A* satisfying

$$ \left|A-\lambda I\right|=\left|\begin{array}{cc}\hfill \left(1-\varphi \right)-\lambda \hfill & \hfill 0\hfill \\ {}\hfill \mu \varphi \hfill & \hfill -\mu -\lambda \hfill \end{array}\right|=0. $$

The corresponding eigenvalues are *λ*_1,2_ = (−*μ*, (1 − *φ*)). This means that the steady state $\left(\widehat{u},\widehat{v}\right)$*=* (0, 1) is stable if *φ* > 1 and unstable if *φ* < 1.

For the fourth steady states, we have matrix,

$$ \left|A-\lambda I\right|=\left|\begin{array}{cc}\hfill \left[1-2\left(\frac{1-\varphi }{1-\varphi \psi}\right)-\varphi \left(\frac{1-\psi }{1-\varphi \psi}\right)\right]-\lambda \hfill & \hfill -\varphi \frac{1-\varphi }{1-\varphi \psi}\hfill \\ {}\hfill -\mu \frac{1-\psi }{1-\varphi \psi}\hfill & \hfill -\mu \left[1-2\left(\frac{1-\psi }{1-\varphi \psi}\right)-\psi \left(\frac{1-\varphi }{1-\varphi \psi}\right)\right]-\lambda \hfill \end{array}\right|=0. $$

The eigenvalues in this case are

$$ {\lambda}_{1,2}=\frac{\left(\varphi -1\right)+\mu \left(\psi -1\right)\pm \sqrt{\left[\left(\varphi -1\right)+\mu {\left(\varphi -1\right)}^2\right]-4\mu \left(1-\varphi \psi \right){\left(\varphi -1\right)}^2}}{2\left(1-\varphi \psi \right)}. $$

**Fig. 6:**
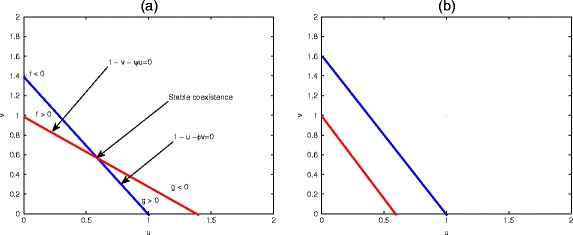
A Lotka-Volterra graph depicting stable equilibrium between two competing species of system (39). Stable coexistence occurs when the isoclines are arranged in (**a**) for *K*_1_ < *K*_2_/*ψ* and *K*_2_ < *K*_1_/*φ*. The intersection point of the two lines gives the positive steady state in (**a**) where the point (1.4, 1.4) corresponds to (1/*φ*, 1/*ψ*). The locations of the isoclines in (**b**) indicate that species u out-competes species v and the point (1/*φ*, 1/*ψ*) corresponds to the value (1.6, 0.6) of species *u* and *v*, respectively

**Fig. 7:**
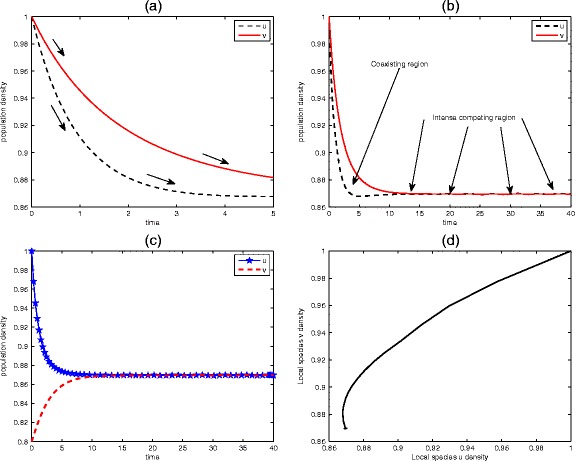
Behaviour of competitive model (38) around the equilibrium states. Declining population density associated with the competitive system is demonstrated in panels (**a**) and (**b**). As the resources declined, the two species compete for the limited resources, as evident in panel (**b**). Parameter values: (**a**) *û* = $\widehat{v}$ =1, *μ* = *φ* = *ψ* = 0.5 at *t* = 5 and (**b**) *û* = $\widehat{v}$ =1, *μ* = 0.5, *φ* = 0.15, *ψ* = 0.15 at *t* = 40. Other parameters are as in (**b**) except at $\widehat{v}$ = 0.8, *t* = 40 for (**c**) and *t* = 20000 for (**d**)

Clearly, the stability of the steady state depends on the size of the positive parameters *μ*, *φ* and *ψ* subject to various cases such as; (*φ* > 1, *ψ* > 1), (*φ* > 1, *ψ* < 1), (*φ* < 1, *ψ* > 1) or (*φ* < 1, *ψ* < 1). A biological interpretation of Fig. 6(b) suggests that because the carrying capacity of species *u* is so high, this species is not limited by the resources to the extent at which species *v* seems to be. Stable coexistence occurs when the isoclines are arranged as in Fig. 6 (a) for *K*_1_ < *K*_2_/*ψ* and *K*_2_ < *K*_1_/*φ*. The populations converge on the intersection of the isoclines regardless of the initial population densities. The intersection point of the two lines gives the positive steady state as in (a) where the point (1.4, 1.4) corresponds to (1/*φ*, 1/*ψ*). The locations of the isoclines in (b) dictate that species *u* out-competes species *v*, the point (1/*φ*, 1/*ψ*) corresponds to the value (1.6, 0.6) of species *u* and *v*, respectively. Clearly, on rearranging, we can see that *ψ* < *K*_2_/*K*_1_ and *φ* < *K*_1_/*K*_2_, and these competition coefficients must be made as small as possible relative to the ratio of its carrying capacity to that of other species. These conditions must hold for both species simultaneously, and this is possible only if the carrying capacities of the two species are similar in such a way that their ratio is close to one. Figure 7 (a) describes the species declining population density associated with the competitive system (38), panels (b,c) refer to the time series solution, and (d) corresponding to the species phase plane diagram.

#### Two dimensional results for model (38)

We also carry out a two-dimensional numerical simulations of the spatially extended competitive model (38). We employed the initial conditions (35) and the zero-flux boundary conditions on a square domain size of [0, 250] × [0, 250] with time-step *Δt =* 0.005 and grid width *Δh =* 0.25. Here the parameter values are set as

$$ \delta =0.05,\kern0.36em \varphi =0.2,\kern0.36em \psi =0.69,\kern0.36em \mu =0.01. $$

**Fig. 8:**
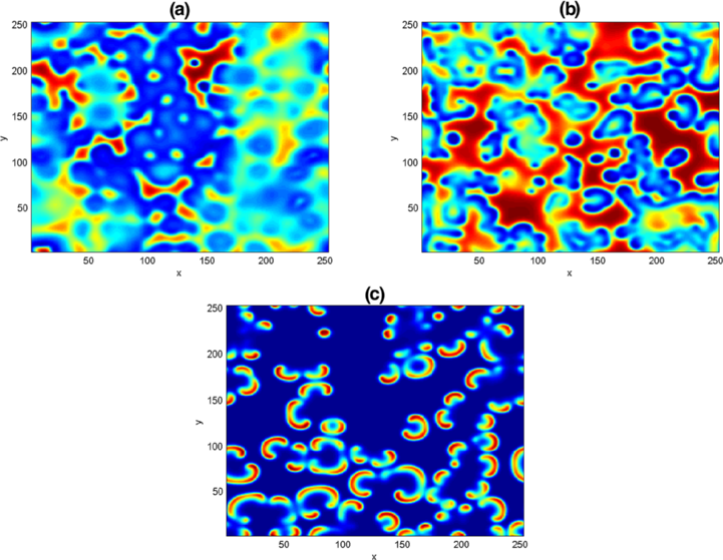
Two dimensional results of the competitive model (38). The patterns are obtained with parameters *t* = 300, *t* = 500 and *t* = 700. Other parameters are as fixed in (42)

In Fig. 8, we show three typical Turing patterns obtained at (a) $\left(\widehat{u},\widehat{v}\right)$*=*(11/45, 110/253) for *t* = 300 and (b) $\left(\widehat{u},\widehat{v}\right)$*=*(0.05, 0.062) for *t* = 500. In both panels, we noticed the formation of Turing spots pattern emanating from the center of the domain, as a result, we fixed the parameter values as in (b) and increase the simulation time to *t* = 700. A pattern containing the mixture of spots and moon-like stripe patterns emerged in (c). From (a-c) one can observed that irregular patterns prevail in the entire domain. However, the three patterns are essentially different from one another, because of their different wavelengths. We believe the possibility of getting other Turing dynamical structures depending on the choice of initial data and the length of simulation.

### Mutualism system

This is a type of association in theoretical ecology in which the existence of one species has no negative influence on the other. This type of model receives little attention and has not been studied as others, even though its importance is comparable to that of prey-predator and competition models. To start with, we shall analyze briefly the two-species model

43 $$ \left.\begin{array}{l}\frac{\partial U}{\partial T}={\sigma}_1\frac{\partial^2U}{\partial {X}^2}+F\left(U,V\right),\\ {}\frac{\partial V}{\partial T}={\sigma}_2\frac{\partial^2V}{\partial {X}^2}+G\left(U,V\right),\end{array}\right\} $$

where *F*(*U*, *V*) = *α*_1_*U*(1 − *U*/*K*_1_ + *β*_1_*V*/*K*_1_) and *G*(*U*, *V*) = *α*_2_*U*(1 − *V*/*K*_2_ + *β*_2_*U*/*K*_2_) are the nonlinear reaction terms for the two species U and V, respectively. And *σ*_1_, *σ*_2_, *α*_1_, *α*_2_, *β*_1_, *β*_2_, *K*_1_, *K*_2_ are all positive parameters. This system looks similar to equation (36), with exception that *β* ' *s* are treated positive in this case. We then nondimensionalize using the parameters

44 $$ u(t)=\frac{U(T)}{K_1},\kern0.24em v(t)=\frac{V(T)}{K_2},\kern0.24em t={\alpha}_1T,\kern0.24em \mu =\frac{\alpha_2}{\alpha_1},\kern0.24em \varphi ={\beta}_2\frac{K_2}{K_1},\kern0.36em \psi ={\beta}_1\frac{K_1}{K_2},\kern0.36em \sigma =\frac{\sigma_2}{\sigma_1}, $$

which on substitution in (43) yields

45 $$ \left.\begin{array}{l}\frac{\partial u}{\partial t}=\frac{\partial^2u}{\partial {x}^2}+\left(u-{u}^2+\varphi uv\right)=f\left(u,v\right),\\ {}\frac{\partial v}{\partial t}=\sigma \frac{\partial^2v}{\partial {x}^2}+\mu \left(v-{v}^2+\psi uv\right)=g\left(u,v\right).\end{array}\right\} $$

Again, by following the linear stability analysis, we study the stability criteria for the non-diffusive system

46 $$ \left.\begin{array}{l}\frac{du}{\partial t}=\left(u-{u}^2+\varphi uv\right)=f\left(u,v\right),\\ {}\frac{dv}{\partial t}=\mu \left(v-{v}^2+\psi uv\right)=g\left(u,v\right),\end{array}\right\} $$

It is not difficult to see that the steady states $\left(\widehat{u},\widehat{v}\right)$ for this system are

47 $$ \left(\widehat{u},\widehat{v}\right)=\left(0,0\right),\kern0.24em \left(\widehat{u},\widehat{v}\right)=\left(1,0\right),\kern0.36em \left(\widehat{u},\widehat{v}\right)=\left(0,1\right),\kern0.36em \left(\widehat{u},\widehat{v}\right)=\left(\frac{1+\varphi }{1-\varphi \psi },\frac{1+\psi }{1-\varphi \psi}\right). $$

The Jacobian or community matrix for this system is

48 $$ B={\left(\begin{array}{cc}\hfill \frac{\partial f}{\partial u}\hfill & \hfill \frac{\partial f}{\partial v}\hfill \\ {}\hfill \frac{\partial g}{\partial u}\hfill & \hfill \frac{\partial g}{\partial v}\hfill \end{array}\right)}_{\left(\widehat{u},\widehat{v}\right)}={\left(\begin{array}{cc}\hfill 1-2u+\varphi v\hfill & \hfill -\varphi u\hfill \\ {}\hfill -\mu \psi v\hfill & \hfill \mu \left(1-2v+\psi u\right)\hfill \end{array}\right)}_{\left(\widehat{u},\widehat{v}\right)}. $$

**Fig. 9:**
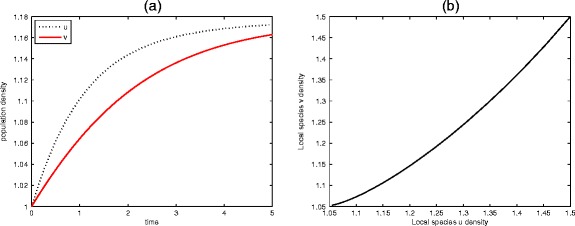
Time series and phase trajectory plane of mutualism system (46). The mutual relationship between the species is shown in panel (**a**), and their phase trajectory in (**b**) indicating linear existence. The parameter values are: *u*_0_ = *v*_0_ = 1, *μ* = 1/2, *φ* = *ψ* = 0.15 at *t* = 5

Proceeding in a similar manner like those for the previous cases, we can easily show that the points (0, 0), (1, 0) and (0, 1) are all unstable; the point (0, 0) is unstable node while (1, 0) and (0, 1) are the saddle point equilibria, whereas the fourth steady state for 1 − *φψ* > 0 (located in the positive quadrant) is a stable equilibrium. Mutual display of the species is reflected in Fig. 9, panel (a) shows linear behaviour of species *u* and *v*. Each of the species experienced an unbounded population growth since the existence of one has no effect on the other and their relationship is linear as in (b).

#### Two dimensional results for model (45)

**Fig. 10:**
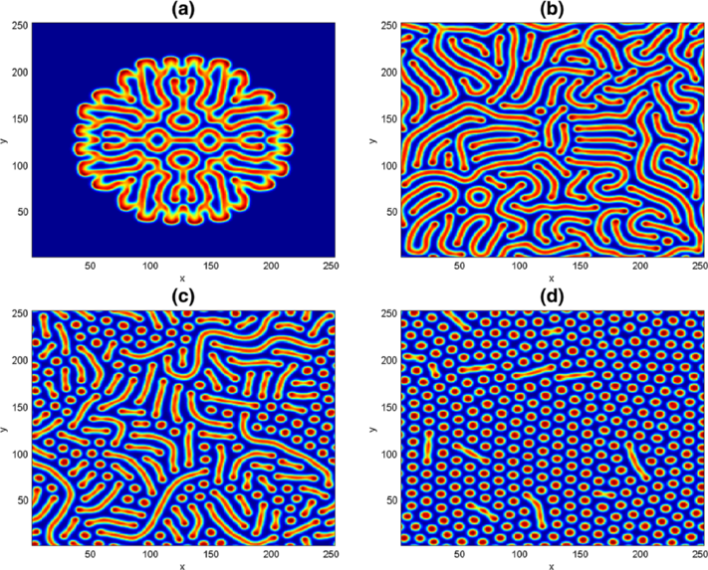
Two dimensional results for the mutualism system (46). The patterns are obtained for panels (**a**-**d**) at *t* = 500, *t* = 1000, *t* = 1500 and *t* = 2000 respectively

Following [34], we take the boundary conditions

49 $$ {\left(\frac{\partial u}{\partial t}\right)}_{\left(x,y\right)}={\left(\frac{\partial v}{\partial t}\right)}_{\left(x,y\right)}=0, $$

subject to the axi-symmetric initial conditions

50 $$ \left.\begin{array}{l}u\left(x,y,0\right)=\widehat{u}-0.5{e}^{\frac{-{\varsigma}^2}{20}},\\ {}v\left(x,y,0\right)=\widehat{v}{e}^{\frac{-{\varsigma}^2}{20}},\end{array}\right\} $$

where *ς*^2^ = *x*^2^/2 + *y*^2^. We perform some numerical simulations of the dynamical model (45) on the domain size [0, 250] × [0, 250] with time-step *Δt* = 0.05 and grid width *Δh* = 0.5, $\left(\widehat{u},\widehat{v}\right)$ =(0.06125, 0.25). We fixed other parameters as in (42) to obtain Fig. 10. In the simulations at *t* = 500, the pattern structures start appearing like a cluster of stripes right from the domain center. It spreads out into irregular stripes as simulation time increased to *t* = 1000. Later, with further increase in time, the long stripes break into spots at *t* = 1500 as in (c). In panel (d) at *t* = 2000, spot patterns have covered the entire domain. Pure Turing spots pattern is achievable if the simulation time is further increased.

In order to justify the suitability and accuracy of the ETDADAMS4 and ETDRK4 schemes, we carried out numerical experiments on the three dynamical systems considered in this paper that is, the prey–predator system (28), competitive system (39), and the mutualism or symbiosis system (45). The performance of ETDRK4 and ETDADAMS4 are investigated and compared with the family of exponential time differencing multisteps schemes of order four, five and six which we denoted in this paper for brevity as ETDM4, ETDM5 and ETDM6 respectively.

It would go beyond the scope of this paper to give a complete classification of exponential integrators used for comparison. We focus on exponential time differencing method of Adams-type and exponential time differencing Runge-Kutta method, and we have mentioned earlier how they can be treated in the common framework of explicit exponential integrators. Details of these schemes are well classified in [16, 41] and references therein, with historical survey offered by Minchev and Wright [30].

We report the maximum relative errors of the solution defined by

51 $$ relative\kern0.24em  error=\frac{{ \max}_{1\le j\le N}\left|{\widehat{u}}_j-{u}_j\right|}{{ \max}_{1\le j\le N}\left|{\widehat{u}}_j\right|} $$

where *û*_j_ is a gold-standard run computed with the schemes at *Δt* = 1/2048 and *u*_j_ is computed values of the solution u at point *j*, and *N* is the number of interior points defined on the collocation interval

52 $$ \left\{{x}_1=a,\dots, {x}_i=a+\left(i-1\right)\varDelta x,\dots, {x}_N=b\right\},\kern0.72em \varDelta x=\frac{\left|b-a\right|}{N-1}. $$

**Fig. 11:**
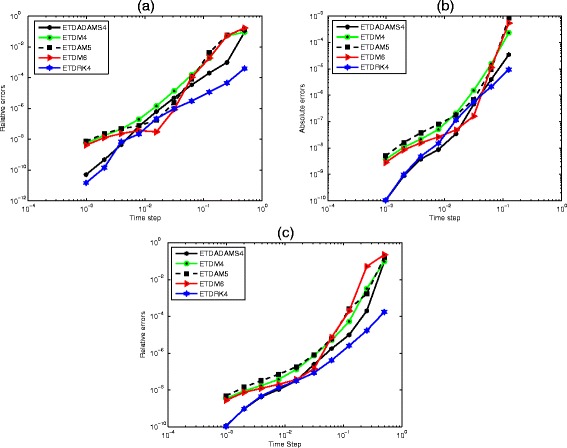
Performance of the schemes (ETDRK4, ETDADAMS4, ETDM4, ETDM5 and ETDM6). Showing accuracies versus time steps for the predator-prey (28), competitive (39) and mutualism (45) models

Figure 11 (a) shows the performance of the schemes when applied to the prey-predator system (28) at parameter values *t =* 1, *μ* = 0.1, *ψ* = 0.08, *φ* = 0.01, *δ* = 0.01 for *N* = 200. Panel (b) is obtained with parameters *t* = 1, *μ* = 0.5, *ψ* = 0.15, *φ* = 0.15, *δ* = 0.5 and *N* = 200 for the competitive system (39). The performance of the schemes when applied to the mutualism system (45) at parameter values t = 1, *μ* = 0.5, *ψ* = 0.5, *φ* = 0.5, *δ* = 0.1, *N* = 200 is shown in panel (c). We compute the relative errors using a gold-standard run obtained with the schemes using *Δt* = 1/2048 and compare with various time steps 1/2^*ρ*^, *ρ* = 1, …, 10 [34, 36].

It is obvious from the results presented in Fig. 11 that the ETDRK4 has a better convergence when compared to other exponential time differencing methods for each of the problems considered in this paper. Due to the similarity and the choices of parameters used in the simulations of the competitive and the mutualism systems, one observes that the schemes have similar behaviour. The difference is noticeable in their amplitudes. The ETDADAMS4 competes very well with ETDRK4 when applied to the dynamical systems but the ETDRK4 appears to have the overall credit.

The following experiment in Table 1 was performed in one-dimension with predator-prey system (28) in a smaller domain size (0; 100) and the computation was terminated at final time *t* = 1,…, 4. The parameter values are: *μ* = 0.4, *ψ* = 0.08, *φ* = 0.05, *Δt* = 0.25 for *N* = 200. We use the built-in Matlab *tic - toc* to check the computational time of the schemes. Both schemes runs in seconds. Our numerical experiments in one-dimension demonstrate a strong case for abandoning the ETDM4, ETDM5 and ETDM6 schemes. In obtaining the 2D results in Fig. 5, it was observed that the ETDRK4 time-stepping scheme performed about two times faster than the ETDADAMS4 scheme. That is, the computational time required for ETDADAMS4 is about 48 % more than that of the ETDRK4. As a result, we carried out the 2D experiments with the ETDRK4 scheme.

**Table 1:** The computational time for the ETDRK4 and ETDADAMS4 methods when applied to the predator-prey system (28) for some values of *δ* and final time *t*

Method	*δ*	*t* = 1	*t* = 2	*t* = 3	*t* = 4
ETDRK4	0.25	0.32 s	0.37 s	1.01 s	0.98 s
	0.50	1.04 s	0.99 s	1.11 s	1.02 s
	1.00	1.15 s	1.32 s	1.88 s	3.87 s
ETDADAMS4	0.25	0.28 s	0.54 s	1.52 s	1.63 s
	0.50	1.51 s	1.50s	1.49 s	1.55 s
	1.00	1.70s	1.83 s	2.51 s	4.40s

## Conclusions

In this paper, firstly, the dynamic complexities of the ecological models consisting of prey-predator, competitive and mutualism reaction-diffusion dynamics are studied by considering their linear stability analysis in the absence of diffusion, and secondly by the numerical approach with the presence of diffusion. We discretized the governing models in space using a fourth-order central finite difference scheme and integrate the resulting ODEs with the exponential time differencing schemes whose formulations were based on the Runge-Kutta and multistep methods of Adams-type. We investigate the stability of the schemes and plots their stability regions. We present the results in both one and two dimensions to unveil their pattern formations. The numerical experiments in 2D reveal some of the typical patterns such as stripes and spots, as well as irregular snakelike patterns. Further, we compared the results obtained with both ETDADAMS4 and ETDRK4 for each of the dynamics, with their exponential fourth, fifth and sixth-orders counterparts denoted as ETDM4, ETDM5 and ETDM6, respectively, and observed that the ETDRK4 is most reliable and computationally promising in terms of efficiency and accuracy when compared to other methods used in this paper. It worth mentioning that the methodology presented in this work can be extended to higher dimensional practical problems.

## Abbreviations

ETDRK4: Fourth-order exponential time differencing Runge-Kutta method
ETDM: Exponential time differencing multistep method
ETDADAMS: Exponential time differencing method of Adams-type
